# Mechanical Behaviour of Completely Decomposed Granite Soil with Tire Rubber Granules and Fibres

**DOI:** 10.3390/polym13234261

**Published:** 2021-12-06

**Authors:** Ru Fu, Wei Li

**Affiliations:** Faculty of Engineering, China University of Geosciences, Wuhan 430074, China; furu@cug.edu.cn

**Keywords:** soil–rubber mixture, compaction, permeability, compressibility, shear strength

## Abstract

Mixing soil with waste tire rubber granules or fibres is a practical and promising solution to the problem of global scrap tire pollution. Before successful applications, the mechanical behaviour of the soil–rubber mixture must be thoroughly investigated. Comprehensive laboratory studies (compaction, permeability, oedometer and triaxial tests) were conducted on the completely decomposed granite (CDG)–rubber mixtures, considering the effects of rubber type (rubber granules GR1 and rubber fibre FR2) and rubber content (0–30%). Results show that, for the CDG–rubber mixture, as the rubber content increases, the compaction curves become more rubber-like with less obvious optimum moisture content. The effect on permeability becomes clearer only when the rubber content is greater than 30%. The shape effect of rubber particles in compression is minimal. In triaxial shearing, the inclusion of rubber particles tends to reduce the stiffness of the mixtures. After adding GR1, the peak stress decreases with the increasing rubber content due to the participation of soft rubber particles in the force transmission, while the FR2 results in higher peak stress especially at higher rubber contents because of the reinforcement effect. For the CDG–GR1 mixture, the friction angle at the critical state (*φ*’*_cs_*) decreases with the increasing rubber content, mainly due to the lower inter-particle friction of the CDG–rubber interface compared to the pure CDG interface, while for the CDG–FR2 mixture, the *φ*’*_cs_* increases with the increasing rubber content, again mainly due to the reinforcement effect.

## 1. Introduction

More than 2 billion units of waste tires are produced globally, and this is expected to increase by 2% every year [1]. The accumulation of waste tires occupies much valuable land space and may also lead to serious safety and environmental problems, such as a high fire risk and the breeding of harmful insects. In developed countries such as the U.S. and Japan, most waste tires are burnt as fuel, and it is reported that waste tires have an even higher calorific value than coal. On the other hand, in developing countries such as China and India, material recovery from waste tires (mainly rubber powder) is the preferred choice [2]. However, both the burning and production of the rubber powder are not environmentally friendly and usually require extra energy to clean. More recently, the reuse of waste tires in the form of tire shreds and granulated rubber as new geo-materials or in the form of mixtures with soil has become a popular approach in the construction of civil engineering structures, such as lightweight embankment fill [3,4], lightweight retaining wall backfill [5,6], drainage layers for roads, landfills, and other applications [7,8,9], thermal insulation to limit frost penetration beneath roads, insulating backfill to limit heat loss from buildings and vibration damping layers for rail lines [10,11,12,13,14,15], which have great potential to alleviate the waste tire accumulation problems and are considered environmentally friendly methods [16].

The physical and mechanical properties of scrap tires and tire–soil mixtures, including compaction, hydraulic properties and compression and shearing behaviours, are the basis of these applications, which have initiated much scientific research. Compaction tests have been performed on pure rubber tires, and the reported dry densities range from 0.40 g/cm^3^ to 0.66 g/cm^3^ [17,18], and it was also found that the compaction energy does not affect the dry density much. Edil and Bosscher [19] studied the compaction behaviour of sand–rubber mixtures and found that the unit weight of the mixtures is mainly controlled by the percentage of soil rather than the water content or compaction effort. Regarding the hydraulic property, it was found that the permeability of the mixture increases with the increasing rubber content [17] and tends to decrease with increasing confining pressure [20,21].

Considering the compression behaviour, Lee et al. [22], Kim and Santamarina [23] and Lee et al. [24] tested the sand–rubber mixtures for cases where D_rubber_:D_sand_ ≈ 0.25:1, D_rubber_:D_sand_ ≈ 10:1 and D_rubber_:D_sand_ ≈ 1:1, respectively. A similar pattern of compression behaviour was observed where the compressibility of the sand–rubber mixtures increased with the increasing rubber content. However, the minimum porosity of the sand–rubber mixtures was achieved when the volumetric rubber content was 40% for the case with D_rubber_:D_sand_ ≈ 0.25:1, while for the case with D_rubber_:D_sand_ ≈ 10:1, the minimum porosity was achieved at 60% volumetric rubber content. Fu et al. [25,26] found that the compression resulting from the addition of rubber particles was balanced by less particle breakage of sand particles due to the cushioning effect of the rubber particles, resulting in similar compressibility values of pure sand and sand–rubber mixtures.

Regarding shearing behaviour, there are studies reporting the strength of sand was reduced after the inclusion of granulated rubber particles, which weakens the interlocking between particles [22,27,28,29]. Typical failure envelopes of sand–tire granules mixtures from Youwai and Bergado [27] show that the failure envelopes moved downwards, indicating lower shear strength, upon increasing the rubber content up to 100% by weight. On the other hand, other studies point out that the strength of sand increased after the inclusion of granulated rubber particles [30,31,32,33]. As shown in Anbazhagan et al. [33], the envelopes of sand mixed with granulated rubber particles moved upwards upon increasing the rubber content to 35% by weight, which is similar to the behaviour of the sand–tire chips mixtures. Overall, based on the published results from laboratory tests, there is no agreement on how granulated rubber affects the mechanical behaviour of sand–rubber mixtures, which is worth investigating further.

It should be noted that in most of the previous studies, natural river sand, due to its good engineering properties (high permeability, stiffness and strength), was chosen to mix with the shredded rubber particles as geomaterials. However, many projects are experiencing a shortage of natural sand fill and the sand price has almost quadrupled in the past decade. So, it is essential to find substitutes that are cheap, readily available and environmentally friendly, to ensure sustainable construction. In this study, a well-graded completely decomposed granite (CDG), which is widely distributed in southeast China, was used as a substitute for natural sand to mix with rubber particles. Different from the quartz sand, the CDG soil generally shows compressive behaviour, as its particles are easy to break.

To better apply this composite material in the field, a comprehensive laboratory study was conducted on these CDG–rubber mixtures, including compaction, permeability, oedometer and triaxial tests, mainly considering the effects of rubber type (a granulated and an elongated) and rubber content (0–30%). Special attention was paid to the triaxial shearing behaviour, as the shearing resistance, at the peak or critical state, is a key property to be obtained, which is used to assess the strength performance of CDG–rubber mixtures.

## 2. Materials and Methods

**Figure 1:**
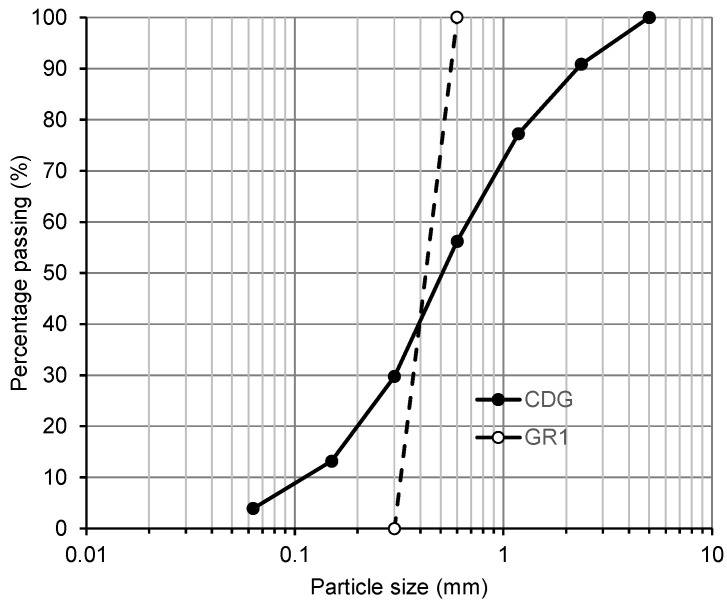
Particle size distributions of CDG and GR1.

**Figure 2:**
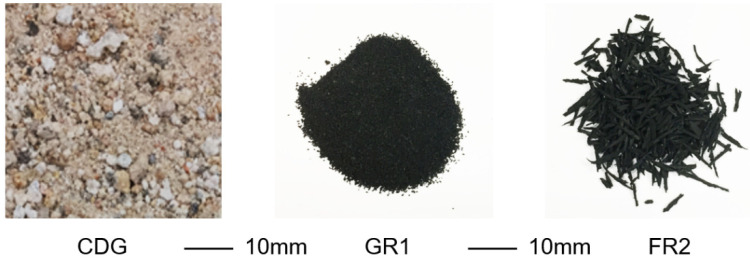
Images of CDG, GR1 and FR2 particles.

For all the tests conducted (compaction, permeability, oedometer and triaxial tests), a well-graded CDG (completely decomposed granite) sand with a specific particle size distribution (C_u_ = 6.3, C_cr_ = 1.2, and D_50_ = 0.51 mm) was used as the host soil. The granulated rubber particles (GR1 (0.3–0.6 mm)), and the fibre type rubber particles (FR2 (average length = 13.48 mm, width = 1.77 mm and aspect ratio = 7.63)) were used in the mixture with CDG. The mixing was performed manually with a metal spoon, and a small amount of water was added to the mixture during mixing to prevent the segregation of rubber particles. Figure 1 shows the particle size distribution curves of CDG and the granulated rubber particles. The size ratios, D_50,rubber_:D_50,CDG_, were about 0.9 for GR1. Considering the length of the fibre-type rubber particles, the size ratios, D_50,rubber_:D_50,CDG_, were about 26.3 for FR2. Figure 2 shows the images of the CDG, granulated rubber and fibre-type rubber particles. The granulated rubber particles were also angular to sub-angular in shape while the fibre-type rubber particles were elongated in shape. The specific gravities (G_s_) were measured following ASTM D854 [34], showing that CDG is 2.65, and rubber particles are 1.15.

**Figure 3:**
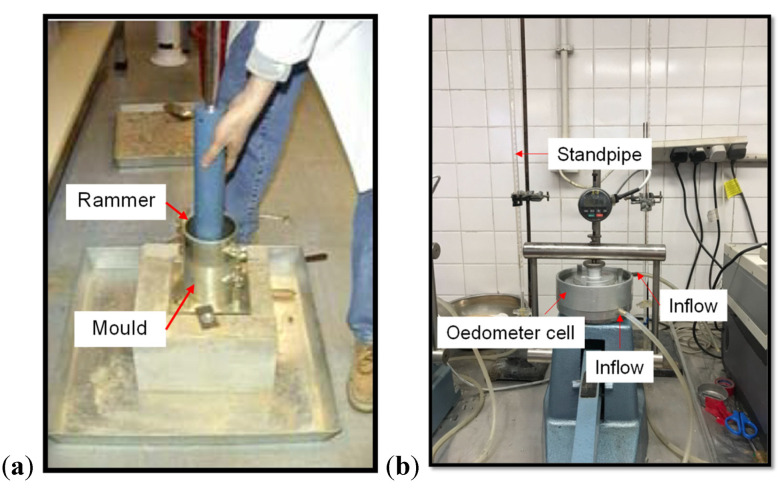

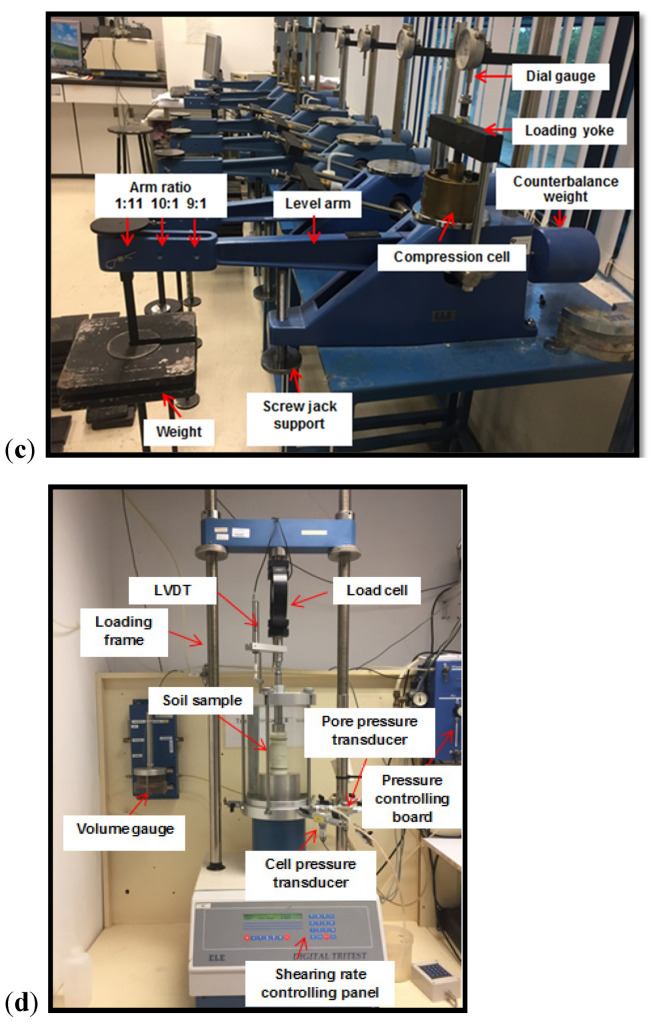
Testing apparatuses: (**a**) Standard Proctor equipment; (**b**) modified oedometer apparatus; (**c**) normal ELE type oedometer apparatus and (**d**) ELE triaxial system.

Compaction tests (standard Proctor test, see Figure 3a) were conducted according to BS1377-4 [35], on pure CDG, pure GR1/FR2 and CDG–rubber mixtures (GR1/FR2) with rubber contents of 10%, 20% and 30%, to obtain relationships between the compacted dry density *ρ_d_* and water content *w* for different materials, in which the corresponding maximum dry density (*ρ_d,max_*) and optimum moisture content (OMC) can be determined. The cylindrical metal mould had internal dimensions of 105 mm in diameter and 115.5 mm in height, and the compaction rammer had a weight of 2.5 kg. The specimen was divided into three layers to compact, and to each layer, 27 blows of the rammer dropping from the controlled height of 300 mm was applied. Note that all the rubber contents mentioned in this study refer to the content by weight, and 30% by weight corresponds to about 50% by volume, above which the mixtures will become rubber dominated [25]. Details of all the compaction tests are given in Table 1.

Falling head permeability tests were performed on pure CDG, pure GR1/FR2 and CDG–rubber (GR1/FR2) mixtures with rubber contents of 10%, 20% and 30%, using a modified oedometer apparatus (see Figure 3b) to investigate effects of rubber shape, content and compression pressure on the permeability of the sand–rubber mixture. The oedometer ring used for the permeability test was 75 mm in diameter and 20 mm in height. A steel rod was used to compact the sample with a predetermined mass at a 90% compaction degree (i.e., 0.9 * *ρ_d,max_*). After compaction, the prepared sample was immersed inside de-aired water for at least 10 h, after which permeability tests were conducted under different vertical stress levels. Details of all the permeability tests are given in Table 2.

For the oedometer tests, a normal ELE-type oedometer apparatus (see Figure 3c) was used for reconstituted samples of pure CDG and CDG–rubber (GR1/FR2) mixtures with rubber contents of 10%, 20% and 30%. Rings with a diameter of 50 mm and a height of 20 mm were used in which the maximum vertical stress that can be reached is approximately 7 MPa. In general, the material was prepared by three layers, with the first layer compacted with less effort and the upper layer with greater effort, to ensure a more uniform density of the sample along its longitudinal axis. Details of all the oedometer tests are given in Table 3.

Consolidated drained triaxial tests were conducted using the ELE triaxial system (see Figure 3d) on both pure CDG and CDG–rubber (GR1 and FR2) mixtures with rubber contents of 10%, 20% and 30%. The specimens were prepared directly on the triaxial base with a membrane attached to a split mould under suction. For pure CDG soil and the CDG–GR1 mixture, the sizes of specimens were 38 mm in diameter and 76 mm in height. For the CDG–FR2 mixture, the sizes of specimens were 60 mm in diameter and 120 mm in height. The compaction procedures were similar to those of the oedometer specimens. Identical compaction energy was adopted for both CDG and CDG–rubber mixture samples to ensure comparability. Details of all the triaxial tests are given in Table 4.

## 3. Engineering Properties of CDG Sand and Its Mixtures with Rubber Particles

### 3.1. Compaction Behaviour

**Figure 4:**
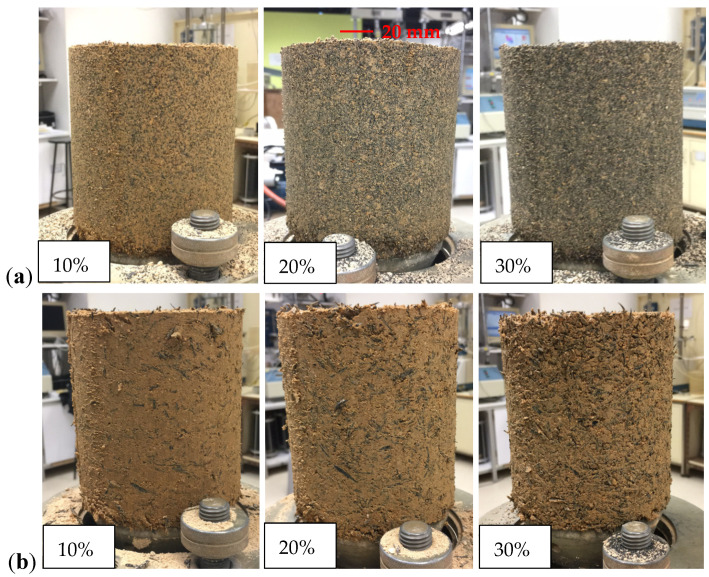
Compaction samples of CDG–rubber mixture at optional moisture content (OMC): (**a**) GR1; (**b**) FR2.

Figure 4 shows the samples of CDG–rubber mixtures compacted at their own optional moisture content (OMC). It can be seen that both the GR1 and FR2 were well mixed with the CDG, and the major difference is that the inclusion of GR1 will further reduce the sand–sand contact due to the much smaller particle size compared with FR2, especially for the higher rubber content. This is consistent with the findings of Lopera Perez et al. [36,37] that the sand–rubber contacts will become dominant after including 30% granulated rubber into the host sand.

**Figure 5:**
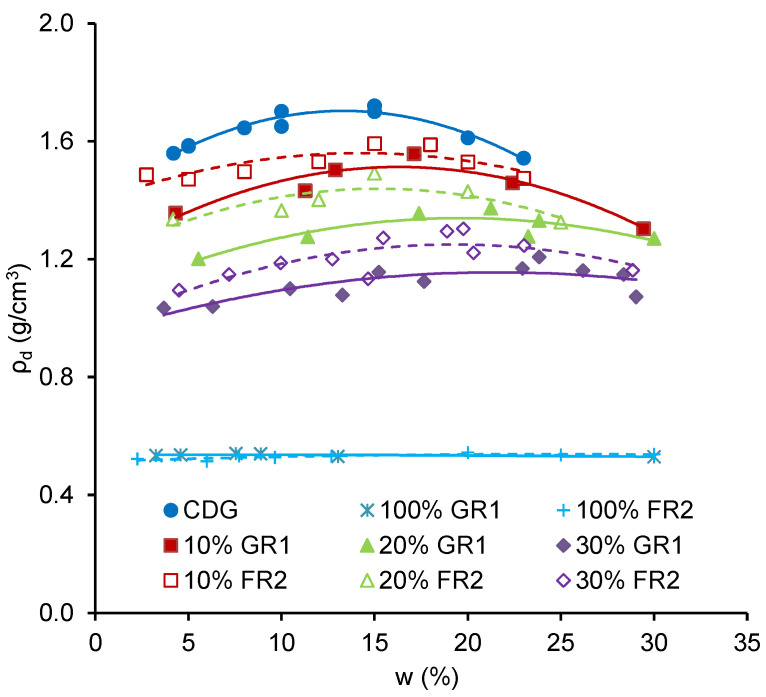
Compaction curves for different mixtures.

**Figure 6:**
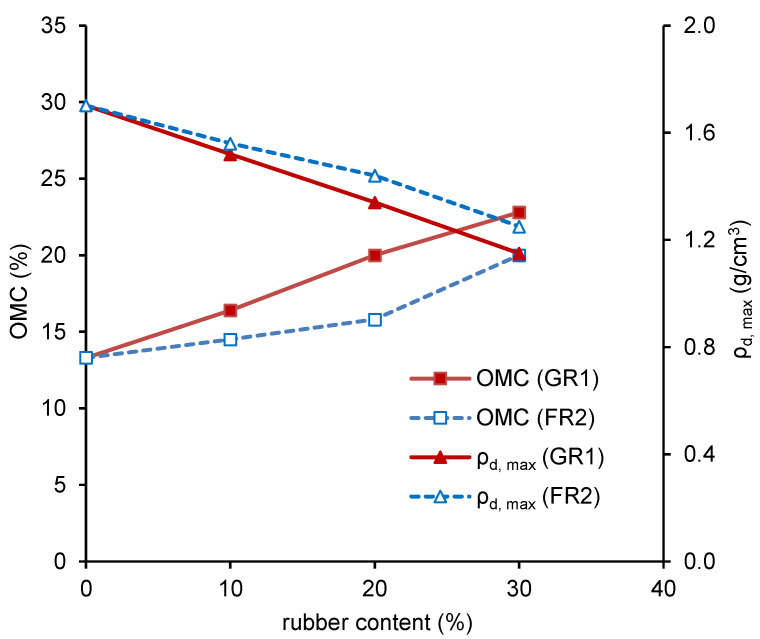
OMC/*ρ_d,max_* against rubber content.

In Figure 5, the dry densities of pure GR1 and FR2 are similar, ranging from 0.51 g/cm^3^ to 0.54 g/cm^3^, and the water content seems not to affect the dry densities much. For the pure CDG, it shows a typical compaction curve, and an obvious OMC can be determined. As the rubber content increases, the compaction curves become more rubber-like with less obvious OMC. In general, the dry density of the CDG–FR2 mixture is higher than that of CDG–GR1 at the given rubber and water contents, which means the elongated FR2 particles are more flexible and work more efficiently in filling the voids of the mixture. Figure 6 shows the effect of rubber content on the OMC and *ρ_d,max_*. It can be seen that OMC increases while *ρ_d,max_* decreases with an increasing rubber content for both GR1 and FR2. Note that the decreasing *ρ_d,max_* here is mainly because of the lower unit weight of the rubber materials.

### 3.2. Permeability

**Figure 7:**
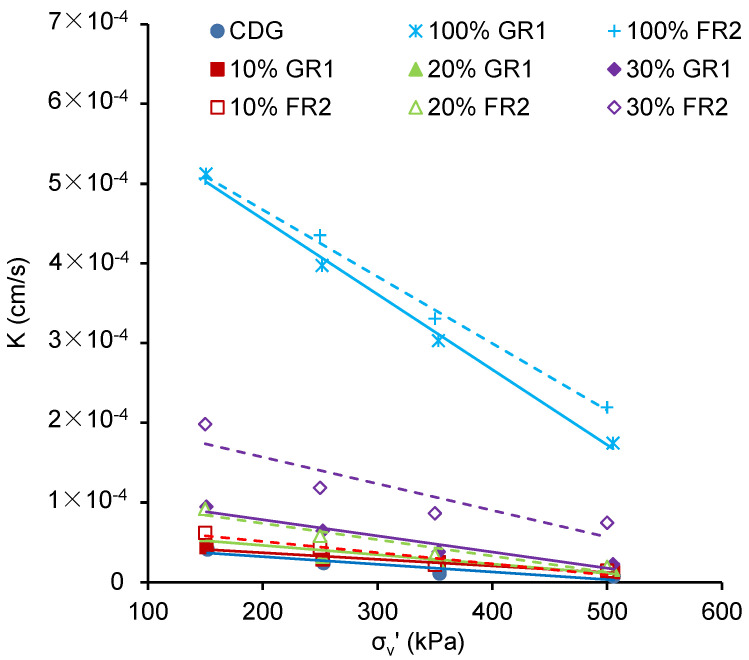
Permeability under increasing compression pressure for CDG–rubber mixtures.

**Figure 8:**
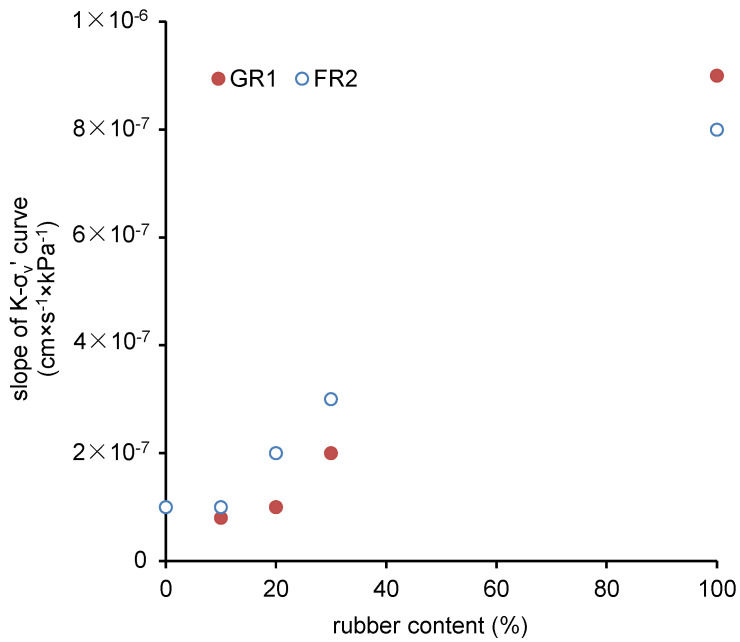
Sensitivity of permeability to pressure for CDG mixed with GR1/FR2.

Figure 7 presents the permeability of those CDG–rubber (GR1/FR2) mixtures under increasing compression pressures. Results show that the permeability of pure GR1/FR2 (5 × 10^−4^ cm/s) is about 10 times higher than that of CDG (4 × 10^−5^ cm/s) at the 150 kPa stress level. The influence on permeability becomes clearer only when the rubber content is greater than 30%. There is a decreasing trend in permeability with an increasing compression pressure. Besides, it can also be observed in Figure 7 that, in a given stress state and rubber content, the CDG–FR2 mixture tends to have higher permeability compared with the CDG–GR1 mixture, even if in previous compaction tests it was shown that the CDG–FR2 is of higher dry density (i.e., lower void ratio). Similarly, the pure CDG has a higher void ratio than those CDG–rubber mixtures, while the permeability of the pure CDG is much lower. A possible reason could be that the CDG–rubber interface is the preferential path for water to permeate. For pure CDG, many of the voids are sealed by fine particles rather than connected as sands, resulting in low permeability. After the inclusion of rubber particles, the sealed voids are connected through those CDG–rubber interfaces. For FR2, due to the larger particle size and elongated particle shape, the CDG–FR2 interface may provide a more connected path for the pore water to permeate. The slope of the permeability–vertical stress curve is adopted as an indicator to assess the sensitivity of permeability of those mixtures to the applied pressure. As shown in Figure 8, with the increasing rubber content, the mixture becomes much more sensitive to the applied pressure.

### 3.3. Compression Behaviour

**Figure 9:**
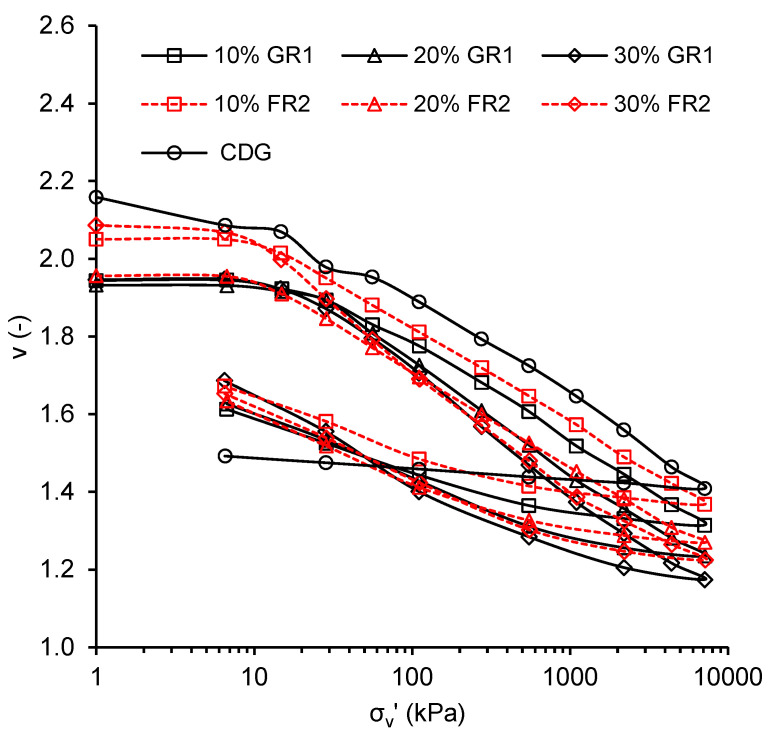
One-dimensional compression curves for CDG–GR1/FR2 mixtures.

Figure 9 shows the one-dimensional compression curves of pure CDG and the CDG–rubber (GR1/FR2) mixtures. The results are quite consistent with previous findings [25]. The inclusion of rubber shifts the compression curve downwards for both GR1 and FR2, while the normal compression line for GR1 is slightly lower than that of FR2. The effect of rubber inclusion in unloading stages is more obvious than that for pure CDG, almost all the volumetric strain is irrecoverable and the strains become much more recoverable with the increasing rubber content for those sand–rubber mixtures, for which the swelling lines are S-shaped.

**Figure 10:**
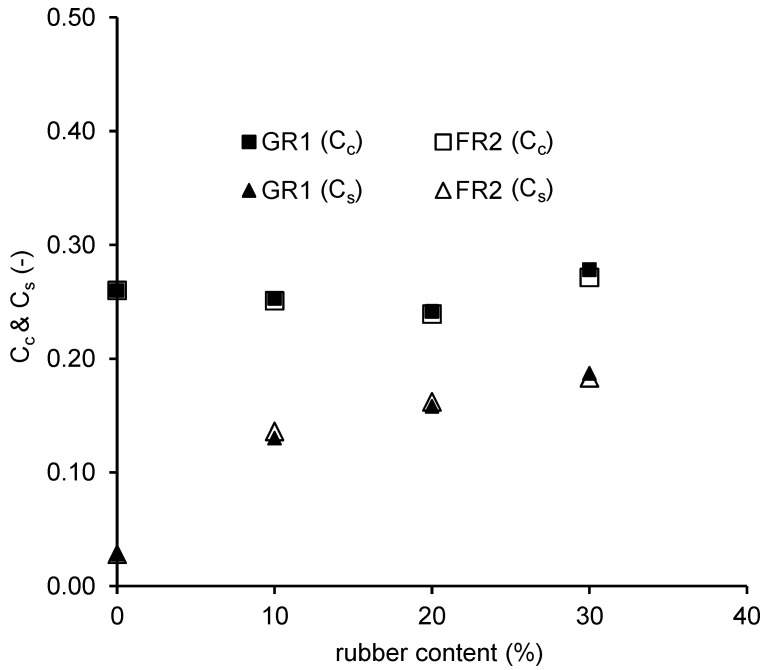
Compression and swelling indices for CDG-GR1/FR2 mixtures.

The compression (C_c_) and swelling indices (C_s_) for all CDG–rubber mixtures are also plotted against the rubber content in Figure 10. For C_s_, the straighter part of the swelling curve (at lower stress levels) was chosen for calculation as performed in Fu et al. [26], since at higher stress levels the elastic deformation of the rubber particles cannot recover, and the mixtures show a much stiffer behaviour. Considering the influence of rubber content, the C_c_ of mixtures with 10–30% rubber content was found to be similar (ranging from 0.233 to 0.328), except a slightly higher compression index at 30% rubber content. This phenomenon can be explained by the combined effects as follows: (i) Less compression due to less particle breakage of CDG particles with the cushioning of rubber particles, and (ii) more compression due to the high deformability of rubber particles. Unlike the compression indices, the C_s_ of mixtures increases obviously with the increasing rubber content, mainly due to the recoverable elastic deformation of rubber particles. Except for the rubber content, the shape of rubber particles seems to have a minimal effect on both the compression and swell indices of CDG–rubber mixtures.

## 4. Shear Behaviour of CDG Mixed with Rubber Particles

### 4.1. Stress–Strain Behaviour

**Figure 11:**
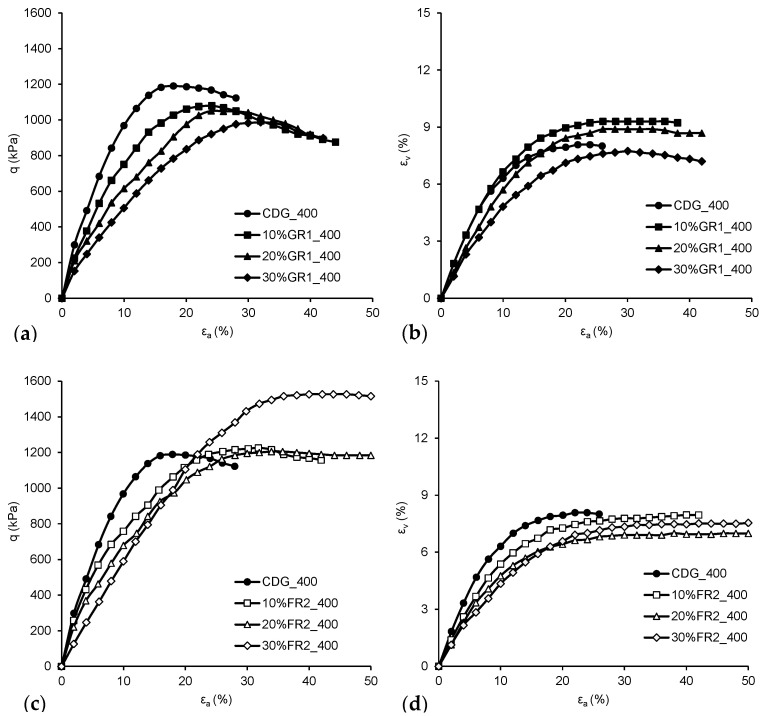
Stress–strain: (**a**) GR1 and (**c**) FR2, and volumetric strain: (**b**) GR1 and (**d**) FR2, behaviour of CDG–rubber mixtures sheared at p’_c_ = 400 kPa (with pure CDG for comparison).

The stress–strain data of the CDG and its mixtures with granulated and fibre-type rubber particles are shown in Figure 11. For clarity, only those specimens sheared at mean effective stress p’_c_ = 400 kPa are presented. From small to moderate strain levels, the stiffness of both mixtures (CDG-GR1/FR2) was reduced with an increasing rubber content because the strong force chain going through softer rubber particles and the tensile strength of the fibre-type rubber particles is not mobilised at such low strain levels (see Figure 11a,c). From moderate to large strain levels, the CDG–GR1 mixtures show slight post-peak strain softening behaviour, with very close stresses at the peak and critical states. The axial strain corresponding to peak stress increases with an increasing rubber content, indicating higher ductility. With the increase in rubber content, the stresses at both the peak and critical states decrease slightly. The CDG–FR2 mixtures show totally different stress–strain behaviour. Even the addition of 10% fibre-type rubber particles does not affect the stress–strain behaviour much, and for mixtures with 20% and 30% rubber particles, higher peak stresses are reached and the peak stress increases with the increasing rubber content, mainly due to the reinforcement effect of those elongated rubber particles. For volumetric strain behaviour, both CDG and its rubber mixtures are nearly purely contractive (see Figure 11b,d). The contraction of CDG mainly comes from the breakage of particles, while the contraction of CDG–GR1 mixtures is attributed to both the breakage of CDG particles and the deformation of rubber particles. The CDG–FR2 mixtures show less contraction than the CDG–GR1 mixtures, which is mainly due to the elongated shape of rubber fibre, making it difficult to fill the voids during shearing.

### 4.2. Shear Strength at the Peak and Critical States

**Figure 12:**
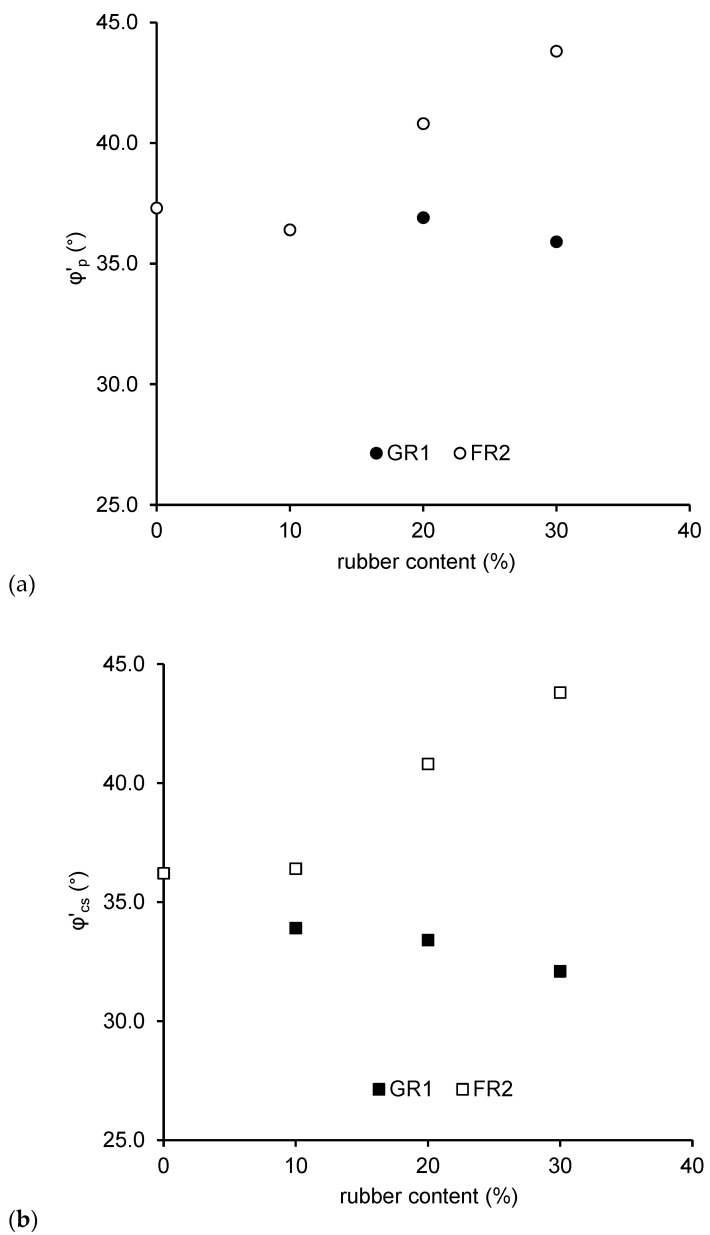
Friction angles at the (**a**) peak states and (**b**) critical states for CDG–GR1/FR2 mixtures.

Figure 12 shows the effect of rubber inclusion on the friction angle at the peak (*φ’_p_*) and critical states (*φ’_cs_*). For CDG, due to the initial loose state, the sample is compressed on shearing until the critical state is reached. In this case, no distinct peak (*φ’_p_* ≈ *φ’_cs_*) will be exhibited. The inclusion of GR1 reduces the *φ’_p_* slightly, since it is unavoidable that the soft rubber particles would participate in the force transmission. Meanwhile, the *φ’_p_* increases when 20% and 30% fibre rubber (FR2) is added due to the mobilised tensile strength of FR2 particles (Figure 12a). Figure 12b shows the effect of rubber inclusion on the *φ’_cs_*. The *φ’_cs_* decreases after adding granulated rubber particles, mainly because the inclusion of rubber leads to less strength mobilised from inter-particle friction as described in Li et al. [38]. The *φ’_cs_* increases after adding fibre rubber particles, again due to the mobilised tensile strength of FR2 particles.

### 4.3. Critical State Lines in V-Lnp’ Space

**Figure 13:**
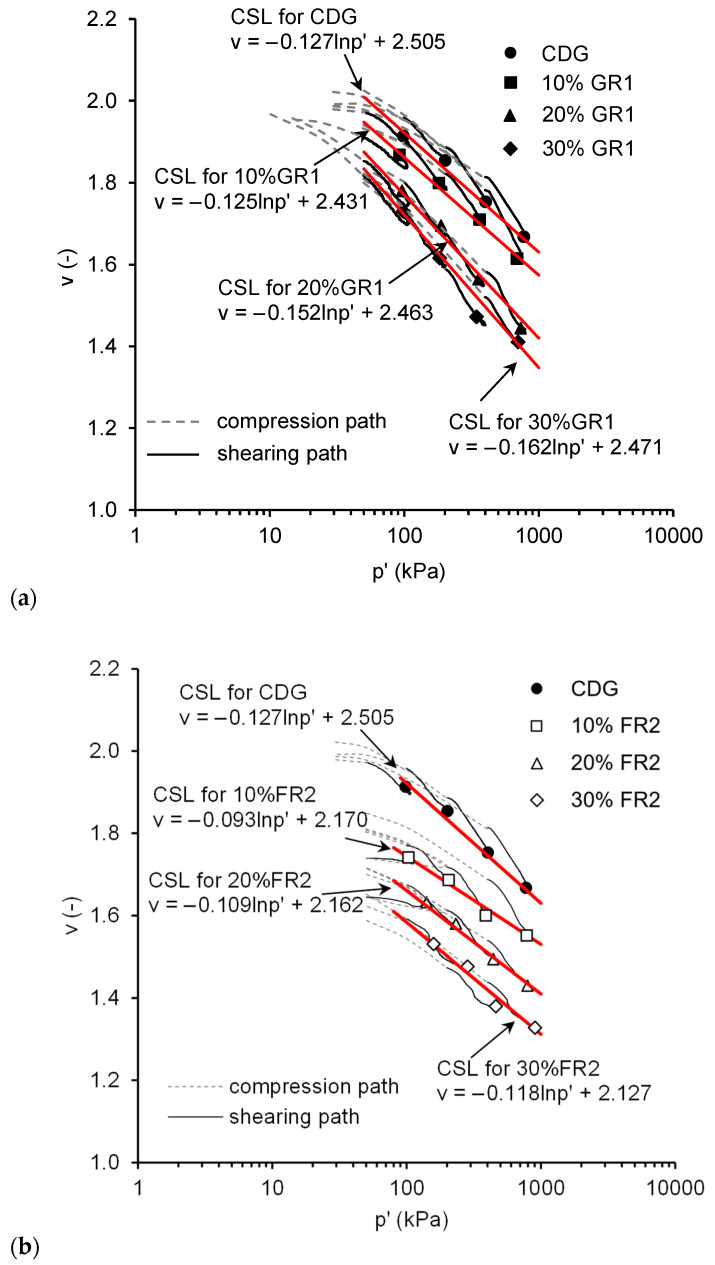
Critical state lines at v-lnp’ plane for CDG–rubber mixtures: (**a**) GR1 and (**b**) FR2.

Figure 13 shows the compression and shearing paths of CDG and its rubber mixtures, where the critical states are marked. It can be seen that a unique CSL can be defined for each material. In Figure 13a, for CDG–GR1 mixtures, the CSL moves downwards when the rubber content increases. The gradient λ, which indicates the compressibility of the material, does not change much when the GR1 content is 10%. As the GR1 content increases to 20% or 30%, λ values are higher, indicating slightly higher compressibility. In Figure 13b, for CDG–FR2 mixtures, a unique CSL can also be defined for each material. Note that those CDG–FR2 samples with higher rubber content that did not reach stable deviatoric stresses would not affect the critical state in the v-lnp’ plane much due to the log scale, so the CSLs obtained are reasonably true. It shows that the CSL also moves downwards with an increasing rubber content, and without significant change in gradient λ. The downwards shift is much more obvious than that of the CDG–GR1 mixture.

### 4.4. Peak Strength with Respect to the State Parameter

**Figure 14:**
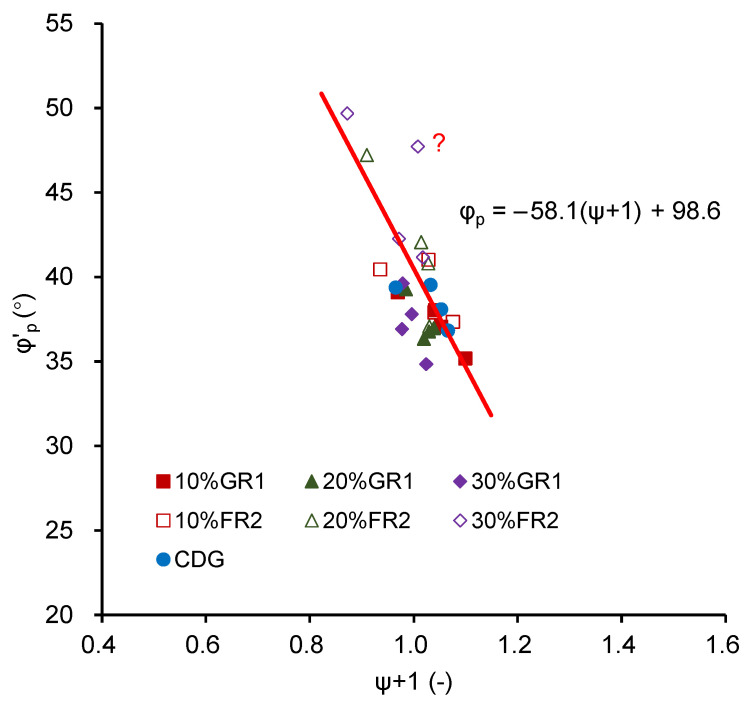
The relationship between peak strength φ’_p_ and state parameter +1 (ψ + 1) for GR1/FR2 mixtures.

The peak strength was discussed with respect to the state parameters proposed by Been and Jefferies [39] in this section. Figure 14 shows the peak strength against state parameters. It can be seen that the peak strength decreases with the increase in state parameters for all the materials tested. Within some scatteredness, a unique relationship can be identified for all the materials, which indicates that the major effect of rubber inclusion in those mixtures is the change of particle packing due to the alteration of the particle size distribution. For CDG–GR1 mixtures, especially at 30% rubber content, the state parameters obviously decrease, indicating denser packing, which results in higher peak strength.

For CDG–FR2 mixtures, the state parameters are clearly reduced, resulting in much higher peak strength. A unique relationship cannot be well defined, especially for the mixtures with 30% FR2, possibly because of the different mechanisms affecting the peak strength of the mixtures. For the mixtures with a higher percentage of elongated rubber particles, the reinforcement effect will become dominant rather than the changing of particle packing as happened in the of CDG–granulated rubber mixtures.

## 5. Summary and Conclusions

This paper provides a comprehensive summary of a series of laboratory tests conducted on a well-graded CDG and the CDG–rubber mixtures, to examine their mechanical properties, including compactability, permeability, compression and shearing behaviour, mainly considering the effects of rubber shape and content. The main points are summarised below:

(1) In Proctor tests, the dry densities of pure rubber particles (both GR1 and FR2) remained constant, ranging from 0.51 g/cm^3^ to 0.54 g/cm^3^, which seems to be independent of water content. For the CDG–rubber mixture, with an increasing rubber content, the compaction curves became more rubber-like with less obvious OMC. The pure rubber particles show about 10 times higher permeability (5 × 10^−4^ cm/s) than that of pure CDG (4 × 10^−5^ cm/s) at the 150 kPa stress level. The influence on permeability becomes clearer only when the rubber content is greater than 30%. With the increasing rubber content, the mixture becomes much more sensitive to the applied pressure.
(2) The inclusion of rubber shifts the compression curve downwards with the increasing rubber content. The C_c_ values of mixtures with 10–30% rubber content are found to be similar, except a slightly higher compression index at 30% rubber content. The effect of rubber inclusion in unloading stages is more obvious when the strains become much more recoverable with the increasing rubber content. The effects of rubber shape are found to be minimal in both compression and unloading stages.
(3) For CDG–granulated rubber mixtures, the stiffness is lower than that of the pure CDG, which is more obvious with the increasing rubber content. The peak strength (*φ’_p_*) decreases slightly with the increasing rubber content. The strength at the critical state (*φ’_cs_*) decreases with the increasing rubber content, which is mainly because the inclusion of rubber leads to less strength mobilised from inter-particle friction. For CDG–rubber fibre mixtures, the stiffness is also lower than that of the pure CDG, indicating that the tensile strength of the fibre-type rubber particles is not mobilised at such low strain levels. In general, both the strengths at the peak and critical states of CDG–rubber fibre mixtures are higher than that of the pure CDG and they increase with the increasing rubber content, mainly due to the reinforcement effect of those elongated rubber fibre particles.
(4) For CDG–granulated rubber mixtures, the inclusion of rubber does not change the state parameters much, so the change of peak strength of those materials is not significant. For CDG–rubber fibre mixtures, the state parameters are clearly reduced resulting in much higher peak strength. For mixtures with a higher percentage of elongated rubber particles, the reinforcement effect will become dominant rather than the change in particle packing as happened in those of CDG–GR1 mixtures.
(5) All the above findings prove the suitability of adding rubber particles to CDG soil as fill materials for various kinds of geotechnical engineering projects, including lightweight fill materials for embankments, drainage layers for roads, landfills and other applications, and reinforced fill materials for retaining walls, which could not only reduce tire waste but also enhance the mechanical behaviour of CDG soil.

## Figures and Tables

**Table 1:** Summary of all compaction tests.

Material Type	Compaction Type	OMC (%)	*ρ_d,max_* (g/cm^3^)
Pure CDG	Standard Proctor test	13.3	1.70
10% GR1		16.4	1.52
20% GR1		20.0	1.34
30% GR1		22.8	1.15
100% GR1		-	0.53
10% FR2		14.5	1.56
20% FR2		15.8	1.44
30% FR2		20.0	1.25
100% FR2		-	0.53

**Table 2:** Summary of all permeability tests using the modified oedometer apparatus.

Test No.	Sand Type	Rubber Type	Rubber Content (%)	Compaction Degree (-)	Vertical Load (kPa)
1	CDG (D_50_ = 0.51 mm, C_u_ = 6.3, C_cr_ = 1.2)	-	0	0.9	50, 150, 250, 350, 500
2		GR1	10	0.9	
3		GR1	20	0.9	
4		GR1	30	0.9	
5		GR1	100	0.9	
6		FR2	10	0.9	
7		FR2	20	0.9	
8		FR2	30	0.9	
9		FR2	100	0.9	

**Table 3:** Summary of all standard oedometer tests.

Material Type	Initial Specific Volume (-)	Maximum Vertical Stress (kPa)
Pure CDG	2.160	7000
10% GR1	1.944	7000
20% GR1	1.932	7000
30% GR1	1.840	7000
10% FR2	2.051	7000
20% FR2	1.957	7000
30% FR2	2.086	7000

**Table 4:** Summary of all standard triaxial tests.

Sand Type	Rubber Type	Characteristics	Rubber Content (%)	Confining Pressure (kPa)
CDG (D_50_ = 0.51 mm, C_u_ = 6.3, C_cr_ = 1.2)	GR1	D_50_ (0.45 mm)	0, 10, 20, 30	50, 100, 200, 400
	FR2	L (13.48 mm) W (1.77 mm) AR (7.62)	10, 20, 30	50, 100, 200, 400

## Data Availability

Data are available by the corresponding author after reasonable request.

## List of Abbreviations

C_c_	compression index
C_cr_	coefficient of curvature
CDG	completely decomposed granite
C_s_	swelling index
C_u_	coefficient of uniformity
D_50_	mean particle size
FR	rubber fibre
GR	rubber granules
G_s_	specific gravity of soil grains
OMC	optimum moisture content
*p’*	mean effective stress
*q*	deviatoric stress
*v*	specific volume
λ	slope of normal consolidation line
*ρ_d_*	dry density
*ρ_d,max_*	maximum dry density
*φ’_cs_*	friction angle at the critical state
*φ’_p_*	friction angle at the peak
ψ	state parameter
Г	specific volume of soil at critical state with p’ = 1.0 kN/m^2^

## References

[1] WBCSD—World Business Council for Sustainable Development (2019). Global ELT management—A Global State of Knowledge on Regulation, Management Systems, Impacts of Recovery and Technologies. https://docs.wbcsd.org/2019/12/Global_ELT_Management%E2%80%93A_global_state_of_knowledge_on_regulation_management_systems_impacts_of_recovery_and_technologies.pdf.

[2] Sienkiewicz M., Kucinska-Lipka J., Janik H., Balas A. (2012). Progress in used tyres management in the European Union: A review. Waste Manag..

[3] Edinçliler A., Baykal G., Saygılı A. (2010). Influence of different processing techniques on the mechanical properties of used tires in embankment construction. Waste Manag..

[4] Soleimanbeigi A., Edil T.B. (2015). Compressibility of recycled materials for use as highway embankment fill. J. Geotech. Geoenvironmental Eng..

[5] Ahn I.-S., Cheng L. (2014). Tire derived aggregate for retaining wall backfill under earthquake loading. Constr. Build. Mater..

[6] Reddy S.B., Krishna A.M. (2015). Recycled tire chips mixed with sand as lightweight backfill material in retaining wall applications: An experimental investigation. Int. J. Geosynth. Ground. Eng..

[7] Narejo D., Shettima M. (1995). Use of recycled automobile tires to design landfill components. Geosynth. Int..

[8] Hudson A.P., Beaven R.P., Powrie W., Parkes D. (2017). Hydraulic conductivity of tyres in landfill drainage systems. Proc. Inst. Civ. Eng. Waste Resour. Manag..

[9] Reddy K., Stark T.D., Marella A. (2008). Clogging potential of tire-shred drainage layer in landfill cover systems. Int. J. Geotech. Eng..

[10] Taylor A.W., Igusa T. (2004). Primer on Seismic Isolation.

[11] Tsang H.-H. (2008). Seismic isolation by rubber–soil mixtures for developing countries. Earthq. Eng. Struct. Dyn..

[12] Hazarika H., Kohama E., Sugano T. (2008). Underwater Shake Table Tests on Waterfront Structures Protected with Tire Chips Cushion. J. Geotech. Geoenvironmental Eng..

[13] Pitilakis K., Trevlopoulos K., Anastasiadis A., Senetakis K. Seismic response of structures on improved soil. Proceedings of the 8th International Conference on Structural Dynamics.

[14] Tsang H.H., Lo S.H., Xu X., Neaz Sheikh M. (2012). Seismic isolation for low-to-medium-rise buildings using granulated rub-ber-soil mixtures: Numerical study. Earthq. Eng. Struct. Dyn..

[15] Tafreshi S.M., Khalaj O., Dawson A. (2014). Repeated loading of soil containing granulated rubber and multiple geocell layers. Geotext. Geomembranes.

[16] Humphrey D.N., Swett M. (2006). Literature Review of the Water Quality Effects of Tire Derived Aggregate and Rubber Modified Asphalt Pavement.

[17] Ahmed I. (1993). Laboratory Study on Properties of Rubber-Soils. Joint Highway Research Project, Report No. FHWA/IN/JHRP-93/4.

[18] Humphrey D., Sandford T. Tire chips as lightweight subgrade fill and retaining wall backfill. Proceedings of the Symposium on Recovery and Effective Reuse of Discarded Materials and By-Products for Construction of Highway Facilities.

[19] Pincus H., Edil T., Bosscher P. (1994). Engineering Properties of Tire Chips and Soil Mixtures. Geotech. Test. J..

[20] Chaney R., Demars K., Masad E., Taha R., Ho C., Papagiannakis T. (1996). Engineering Properties of Tire/Soil Mixtures as a Lightweight Fill Material. Geotech. Test. J..

[21] Reddy K.R., Saichek R.E. Characterization and performance assessment of shredded scrap tires as leachate drainage material in landfills. Proceedings of the 14th International Conference on Solid Waste Technology and Management.

[22] Lee J.S., Dodds J., Santamarina J.C. (2007). Behavior of rigid–soft particle mixtures. J. Mater. Civ. Eng..

[23] Kim H.-K., Santamarina J.C. (2008). Sand–rubber mixtures (large rubber chips). Can. Geotech. J..

[24] Lee C., Shin H., Lee J. (2014). Behaviour of sand-rubber particle mixture: Experimental observations and numerical simulations. Int. J. Numer. Anal. Methods Geomech..

[25] Fu R., Coop M.R., Li X.Q. (2014). The mechanics of a compressive sand mixed with tyre rubber. Geotech. Lett..

[26] Fu R., Coop M.R., Li X.Q. (2017). Influence of Particle Type on the Mechanics of Sand–Rubber Mixtures. J. Geotech. Geoenvironmental Eng..

[27] Youwai S., Bergado D.T. (2003). Strength and deformation characteristics of shredded rubber tire—Sand mixtures. Can. Geotech. J..

[28] Neaz Sheikh M., Tsang H.H., Yaghmaei-Sabegh S., Anbazhagan P. Evaluation of damping modification factors for seismic response spectra. Proceedings of the Australian Earthquake Engineering Society Conference.

[29] Noorzad R., Raveshi M. (2017). Mechanical Behavior of Waste Tire Crumbs–Sand Mixtures Determined by Triaxial Tests. Geotech. Geol. Eng..

[30] Ghazavi M. (2004). Shear strength characteristics of sand-mixed with granular rubber. Geotech. Geol. Eng..

[31] Attom M.F. (2006). The use of shredded waste tires to improve the geotechnical engineering properties of sands. Environ. Geol..

[32] Anbazhagan P., Manohar D.R., Neaz Sheikh M. Response surface analysis for engineering behavior of sand-tire crumb mixtures. Proceedings of the 2nd International Conference on Information Technology in Geo-Engineering.

[33] Anbazhagan P., Manohar D.R., Rohit D. (2017). Influence of size of granulated rubber and tyre chips on the shear strength characteristics of sand–rubber mix. Géoméch. Geoengin..

[34] ASTM D854 (2002). Standard Test Methods for Specific Gravity of soil Solids by Water Pycnometer. Annual Book of ASTM Standards.

[35] BSI (1990). Methods of Test for Soils for Civil Engineering Purposes. BS1377.

[36] Perez J.L., Kwok C., Senetakis K. (2016). Effect of rubber size on the behaviour of sand-rubber mixtures: A numerical investigation. Comput. Geotech..

[37] Perez J.L., Kwok C., Senetakis K. (2017). Micromechanical analyses of the effect of rubber size and content on sand-rubber mixtures at the critical state. Geotext. Geomembranes.

[38] Li W., Kwok C., Sandeep C.S., Senetakis K. (2019). Sand type effect on the behaviour of sand-granulated rubber mixtures: Integrated study from micro- to macro-scales. Powder Technol..

[39] Been K., Jefferies M.G. (1985). A state parameter for sands. Géotechnique.

